# The Combination of a Low-Dose Chemotherapeutic Agent, 5-Fluorouracil, and an Adenoviral Tumor Vaccine Has a Synergistic Benefit on Survival in a Tumor Model System

**DOI:** 10.1371/journal.pone.0067904

**Published:** 2013-06-28

**Authors:** Sean M. Geary, Caitlin D. Lemke, David M. Lubaroff, Aliasger K. Salem

**Affiliations:** 1 Division of Pharmaceutics, College of Pharmacy, University of Iowa, Iowa City, Iowa, United States of America; 2 Departments of Urology and Microbiology, Holden Comprehensive Cancer Center, University of Iowa, Iowa City, Iowa, United States of America; University of Chicago, United States of America

## Abstract

Standard cancer therapies, particularly those involving chemotherapy, are in need of modifications that both reduce short-term and long-term side effects as well as improve the overall survival of cancer patients. Here we show that combining low-dose chemotherapy with a therapeutic vaccination using an adenovirus encoding a model tumor-associated antigen, ovalbumin (Ad5-OVA), had a synergistic impact on survival in tumor-challenged mice. Mice that received the combinatorial treatment of Ad5-OVA plus low-dose 5-fluorouracil (5-FU) had a 95% survival rate compared to 7% and 30% survival rates for Ad5-OVA alone and 5-FU alone respectively. The presence of 5-FU enhanced the levels of OVA-specific CD8^+^ T lymphocytes in the spleens and draining lymph nodes of Ad5-OVA-treated mice, a phenomenon that was dependent on the mice having been tumor-challenged. Thus 5-FU may have enhanced survival of Ad5-OVA-treated mice by enhancing the tumor-specific immune response combined with eliminating tumor bulk. We also investigated the possibility that the observed therapeutic benefit may have been derived from the capacity of 5-FU to deplete MDSC populations. The findings presented here promote the concept of combining adenoviral cancer vaccines with low-dose chemotherapy.

## Introduction

Cancer is responsible for one quarter of deaths in the United States and current conventional treatments are proving inadequate at combating the majority of these malignant diseases [1]. Chemotherapy can have dramatic effects at eliminating tumor mass but often tumors will recur if the chemotherapy is not maintained or because of selection-induced resistance [2]. Despite its limited therapeutic success, chemotherapy, for lack of better alternatives, is often the default treatment for many cancer patients. Sustained high doses of chemotherapeutic drugs can cause severe side-effects that include irreversible damage to vital organs and can be themselves carcinogenic [3], [4]. Therefore, alternative or adjuvant therapies are required that are capable of replacing, or reducing the delivery dose of, chemotherapeutic drugs.

Therapeutic cancer vaccinations using viral vectors encoding relevant tumor-associated antigens (TAA) have shown promising therapeutic benefit but are often less successful than expected in clinical settings, possibly due to excessive tumor burden and the presence of host-derived immunosuppressive mechanisms [5], [6]. Recombinant adenovirus type 5 vectors (Ad5) are efficient at transducing genes to a range of cell types, including dendritic cells, and are therefore good candidates for the delivery of TAAs [7], [8], [9]. Aside from being one of the most efficient vectors for gene delivery *in vivo*, Ad5 and other Ad strains (e.g. Ad35) possess an inherent ability to, not only potently infect, but also stimulate dendritic cells [8], [9]. In a mouse model of prostate cancer it was shown that Ad5 encoding human PSA (Ad5-PSA) could induce functionally effective PSA-specific CD8^+^ T lymphocyte responses [10]. It was also shown that vaccination with Ad5-PSA admixed with the collagen matrix, Gelfoam®, resulted in both stronger immune responses and protection from high titer anti-adenovirus antibodies, when compared to Ad5-PSA administered alone [11]. Such a finding is particularly significant since a large percentage of the population is likely to have pre-existing immunity capable of neutralizing Ad5 infectivity. Clinical trials with Ad5-TAA cancer vaccines are few; however, the indications thus far, combined with preclinical data, suggest that supplementary treatments are likely to be necessary to improve the therapeutic potency of Ad5-TAA [5], [10], [11], [12]. This is a situation that not only applies to other viral-based cancer vaccines, but to cancer vaccines in general [13], [14], [15], [16]. A preclinical study recently highlighted the immune and therapeutic benefits of sequentially combining an Ad5 vaccine (encoding HPV-E7) with high-dose chemotherapy (cisplatin and gemcitabine) [17]. However, high-dose chemotherapy does have many undesirable side effects that should ultimately be avoided or circumvented. Studies involving the combination of viral cancer vaccines (encoding TAA) and low-dose chemotherapy are scarce and require investigation.

The concept of combining therapeutic cancer vaccines with limited, or low-dose, chemotherapy has garnered interest partially due to accumulating findings that some chemotherapeutic agents can selectively abrogate the suppressive arm or stimulate the effector arm of the immune response [18], [19]. Most notably, low-dose cyclophosphamide has a significant effect at reducing regulatory T cell (Treg) function [20], [21] and clinical trials combining low-dose cyclophosphamide with non-viral-based cancer vaccines have yielded promising results [18], [22]. More recently, murine studies have demonstrated that another chemotherapeutic drug, 5-fluorouracil (5-FU), when used at low doses can reduce the levels of myeloid-derived suppressor cells (MDSCs) and promote immune-mediated tumor rejection [23]. MDSCs are known to accumulate in tumors, blood and draining lymph nodes of cancer patients, where they exert an immunosuppressive effect via a range of mechanisms, which include the promotion of Treg function [24]. Systemic elimination of MDSCs may prove a more attractive therapeutic approach over systemic Treg depletion due to the reduced potential for pathological autoimmune consequences. Despite accruing interest, there is still a paucity of preclinical and clinical studies that address the benefit of combining viral-based cancer vaccines with low-dose chemotherapy [25].

Here we used a well characterized mouse ovalbumin (OVA)-expressing thymoma cell line (E.G7) often used as a model for tumor immunotherapy where OVA is the model TAA [26]. We chose to combine low-dose 5-FU (40 mg/kg) and Ad5-OVA in an attempt to achieve therapeutic synergy to E.G7-challenged immunocompetent mice. We have previously demonstrated Ad5-OVA alone to be only marginally therapeutic [12]. Results presented here show that the therapeutic combination of Ad5-OVA and 5-FU is significantly more effective at eliminating tumors than either treatment alone. In order to gain mechanistic insight into the therapeutic success we determined relative levels of potentially key immune populations, such as tumor-specific CD8^+^ T lymphocytes, Tregs and MDSCs in the spleens and lymph nodes of treated mice.

## Materials and Methods

### Mice and Cell Lines

C57BL/6 (H2K^b^) male mice between 6–8 weeks of age were obtained from Jackson Laboratories (Bar Harbor, ME) and maintained in filtered cages. E.G7 (thymoma cell line transfected with chick ovalbumin [26] obtained from American Type Culture Collection, Manassas, VA) were grown in RPMI-1640 (GIBCO, Invitrogen, CA) supplemented with 10% fetal bovine serum (Atlanta Biologicals, GA), 1 mM sodium pyruvate (GIBCO, Invitrogen, CA), 10 mM HEPES (GIBCO, Invitrogen, CA), 0.05 mM 2-mercaptoethanol, and 50 µg/ml gentamicin sulfate (Mediatech, Inc., VA) and selection was maintained with 0.4 mg/mL G418 (GIBCO, Invitrogen, CA). All the animal experiments were performed following specific approval by the University of Iowa Institutional Animal Care and Use Committee and in accordance with guidelines and regulations approved by the University of Iowa Institutional Animal Care and Use Committee.

### Adenovirus and 5-FU

Replication-deficient adenovirus type 5 (with E1A and E1B genes deleted) encoding either chicken ovalbumin (Ad5-OVA) or beta-galactosidase (Ad5-LacZ) were generated using standard methods by the University of Iowa Gene Transfer Vector Core (Iowa City, IA) [27]. 5-Fluorouracil (5-FU) was purchased from Sigma (St. Louis, MO).

### Tumor Challenge and Therapeutic Protocol

For tumor challenge, 7–10 week old C57BL/6 mice were anesthetized by intraperitoneally (i.p.) injection of a ketamine/xylazine mix (87.5 mg/kg ketamine; 2.5 mg/kg xylazine) which was purchased from the Office of Animal Resources (University of Iowa, Iowa City, IA). Mice were injected subcutaneously (s.c.) with 10^6^ E.G7 cells into the dorsal right flank. Seven days later, mice were immunized s.c. with 10^8^ pfu (2.7×10^9^ virus particles) of Ad5-OVA ± i.p. administration of 40 mg/kg 5-FU. Tumor outgrowth, determined by tumor size as a function of time, was measured two – three times per week and tumor volume was calculated by the equation for determining the volume of an ellipsoid: [(Diameter 1 × Diameter 2 × Height) × (π/6)], as previously described [28]. Mice were sacrificed when tumors reached a diameter of 20 mm in any direction. The dose of 5-FU, at 40 mg/kg, was designated “low-dose” based on two factors: 1) 5-FU-treated mice showed no observable side effects often associated with “high-dose” chemotherapy (ruffled fur, squinting eyes, loss of appetite, hunched posture): 2) the equivalent dose in humans has been shown to be minimally symptomatic or asymptomatic [29] [30].

### Isolation of Peripheral Blood Lymphocytes (PBLs), Splenocytes and Draining Lymph Node Lymphocytes

PBLs were isolated by submandibular bleeds, red blood cells were lysed using ACK buffer (NH_4_Cl/KHCO_3_/EDTA solution) and the remaining cells were stained for the presence of OVA-specific CD8^+^ T cells using an OVA-tetramer as described below. Lymphocytes were isolated from freshly harvested spleens and draining lymph nodes by homogenization in a small volume of isotonic media. Single cell suspensions were collected by passing the homogenized tissue through a 70 µm cell strainer and then stained for the presence of Tregs, MDSCs or OVA-specific CD8^+^ T cells (see below). Prior to staining the splenocytes were also treated with ACK buffer to lyse red blood cells.

### Staining Cells Isolated from Spleens, Draining Lymph Nodes and Peripheral Blood for the Presence of Tregs, MDSCs or OVA-specific CD8^+^ T cells

The frequency of OVA-specific CD8^+^ T cells was determined by tetramer staining, as previously described [28]. The tetramer used was the H-2K^b^ SIINFEKL Class I iTAg™ MHC tetramer (K^b^-OVA_257_) labeled with PE (Beckman Coulter, Fullerton, CA). Surface CD8 and CD3 were stained with anti-CD8-FITC and anti-CD3-PE-Cy5 mAbs (eBioscience, San Diego, CA) respectively. The levels of OVA-specific CD8^+^ T cells were expressed as a percentage of total CD3^+^CD8^+^ cells in the spleen, draining lymph node or peripheral blood, and were normalized against the results obtained for naïve (untreated) mice when pooled data were used. The frequency of Foxp3^+^CD3^+^CD4^+^ Tregs was determined using a Foxp3 staining kit (eBioscience). The Foxp3 antibody was PE-labeled (eBioscience) and surface CD4 and CD3 were stained with anti-CD4-FITC and anti-CD3-PE-Cy5 mAbs (eBioscience) respectively. The levels of Tregs were expressed as a percentage of total CD3^+^CD4^+^ cells in the spleen or draining lymph node, and were normalized against the results obtained for naïve (untreated) mice when pooled data were used. MDSCs were detected by staining with a combination of anti-CD11b-PE (eBioscience), anti-Ly6C-FITC (BioLegend) and anti-Ly6G-PECy5 (eBioscience). Levels of gMDSCs or mMDSCs were expressed as a percentage of the entire splenocyte or lymph node populations, and were normalized against the results obtained for naïve (untreated) mice when pooled data were used. Samples were acquired using a FACScan flow cytometer (Becton Dickinson, NJ) and analyzed with FlowJo software (TreeStar, OR).

### MDSC Depletion

MDSC depletions were achieved using an anti-GR-1 monoclonal antibody (RB6-8C5) (kindly provided as a hybridoma by Dr. David Sibley (Washington University, St Louis, MO)). Use of RB6-8C5 as a suitable agent for depleting MDSCs from tumor-bearing mice was based on findings by Morales et al (2008) [31]. Briefly, MDSC depletions were commenced on day 6 post tumor challenge by i.p. administration of 300 µg of RB6-8C5. RB6-8C5 was re-administered daily on days 7–12.

### Statistics

All statistical analyses were performed using GraphPad Prism software, version 5.00 for Windows (San Diego, CA). Unless otherwise stated data from pooled experiments were normalized and the ratios generated were analyzed using an ANOVA one way analysis of variance (with a Tukey post test). Analysis of survival curves was performed using log-rank (Mantel-Cox) test.

## Results

### Ad5-OVA Plus 5-FU in Combination Provide Synergistic Therapeutic Benefit to Tumor-challenged Mice

**Figure 1:**
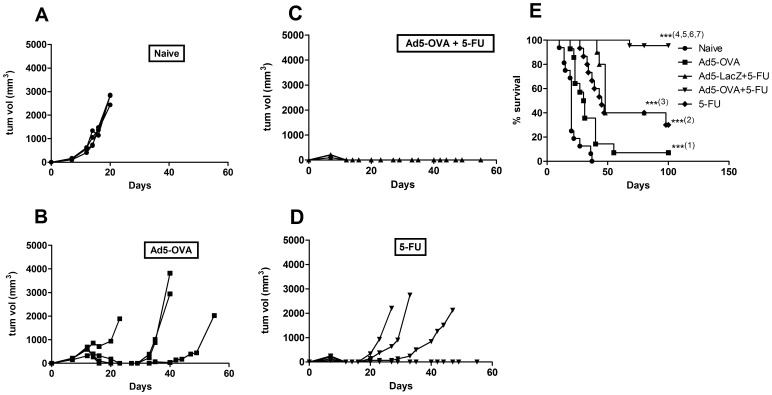
Anti-tumor effect and survival in mice treated with Ad5-OVA and/or 5-FU. C57BL/6 mice were challenged with E.G7 and then, 7 days post tumor challenge, given: no treatment (naïve); Ad5-OVA; Ad5-OVA +5-FU; or 5-FU. *A–D.* The tumor volumes for each mouse from one representative experiment are shown. *E.* Survival graph representing four pooled experiments. Total number of mice for each treatment was: n = 15 for naïve group, n = 14 for Ad5-OVA alone group, n = 21 for Ad5-OVA +5-FU group, n = 10 for Ad5-LacZ +5-FU group, and n = 15 for the 5-FU alone group. Statistical analysis (Log-rank (Mantel-Cox) test) of survival data revealed that mice survived significantly longer, compared to untreated mice, when treated with Ad5-OVA alone (p<0.001 (***^(1)^)), 5-FU alone (p<0.001(***^(2)^)) or Ad5-LacZ +5-FU (p<0.001(***^(3)^), and that mice treated with Ad5-OVA +5-FU in combination survived significantly longer than untreated mice (p<0.001 (***^(4)^) and mice treated with Ad5-OVA alone (p<0.001 (***^(5)^)), 5-FU alone (p<0.001(***^(6)^)) and Ad5-LacZ +5-FU (p = 0.001 (***^(7)^).

Mice challenged with OVA-expressing E.G7 tumor cells were inoculated peritumorally with Ad5-OVA (10^8^ PFU) and/or an injection of 5-FU (40 mg/kg: i.p.) on day 7 post tumor challenge. Mice were monitored for tumor progression and it was found that most or all of the mice in each of the treatment groups initially responded with substantial tumor regression compared to the untreated (naïve) mice. Fig. 1*A–D* shows the tumor volumes for each mouse receiving the indicated treatment from one representative experiment, whilst Fig. 1*E* shows the survival data from four pooled experiments. The naïve group had tumors that progressively grew and these mice usually had to be sacrificed by day 20 post-tumor challenge. Mice treated with Ad5-OVA alone generally experienced tumor regression that commenced approximately 3–5 days post vaccination, and displayed transient remissions. However, most of these mice eventually experienced tumor recurrence with progression and only 7% remained tumor-free. Mice treated with 5-FU alone resulted in faster regressions compared to mice treated with Ad5-OVA alone. Once again, however, only transient remission periods were observed for the majority of mice, with 30% remaining tumor-free. Mice treated with Ad5-LacZ plus 5-FU had tumor regression kinetics (not shown) and survival outcomes (Fig. 1*E*) similar to mice treated with 5-FU alone. In contrast, the treatment group receiving the combination of Ad5-OVA plus 5-FU proved to be significantly more effective long term than Ad5-OVA alone, 5-FU alone or Ad5-LacZ plus 5-FU, since tumors were rapidly eliminated and did not recur for 95% of mice, which were monitored for ≥80 days. The surviving mice from the 5-FU alone group were susceptible to subsequent tumor (E.G7) challenge, whilst the surviving mice from the Ad5-OVA alone and Ad5-OVA plus 5-FU groups resisted subsequent tumor challenge (data not shown). Such findings would suggest that 5-FU alone does not cause rejection of tumors through adaptive immunity whilst vaccination with Ad5-OVA alone or in combination with 5-FU does.

### The Effect of 5-FU on the Generation of Ad5-OVA-induced OVA-specific CD8^+^ T cells in Tumor-challenged Mice and Non-tumor-challenged Mice

**Figure 2:**
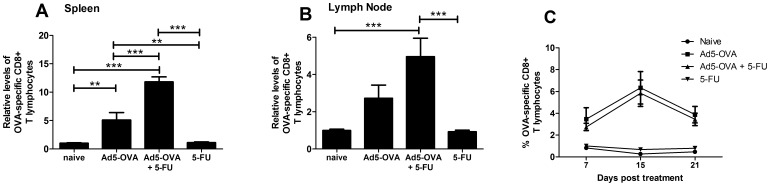
OVA-specific CD8^+^ T cell responses in tumor challenged and non-tumor-challenged mice. C57BL/6 mice were challenged with E.G7 and then, 7 days post tumor challenge, were treated as indicated. *A* and *B*. On day 9 post treatment the levels of OVA-specific CD8+ T cells, as a percentage of total CD8+CD3+ cells, were determined using a fluorescently-tagged OVA tetramer (see materials and methods for further details) in samples from the spleen (*A*) and the lymph node (*B*). The percentages were normalized against the mean values obtained for the naïve groups. Mean percentage values for the naïve group prior to normalization were 0.26% (range 0.16–0.39%) for the lymph node and 0.68% (range 0.54–0.89%) for the spleen. Data represents pooled data from four separate experiments, where the number of mice/treatment group in each experiment was n = 1, n = 2, n = 2 and n = 4 and therefore a total of n = 9 mice from each pooled treatment group were analyzed. Statistical significance was determined using an ANOVA one way analysis of variance (with Tukey post-test) (**P<0.01, ***P<0.001). *C* **.** Non-tumor-challenged mice (n = 4 per group) were vaccinated with indicated treatments and then the levels of OVA-specific CD8^+^ T cells in the peripheral blood were measured on days 7, 15 and 21. These results were shown to be reproducible. Error bars represent standard error of the mean.

On day 9 post-treatment, tumor-challenged mice from each treatment group (described above) were sacrificed and cells from the spleen and draining lymph node were stained for the presence of OVA-specific CD8^+^ T cells**.** Fig. 2 shows the results obtained from four pooled independent experiments. There was a highly significant increase in the proportion of OVA-specific CD8^+^ T cells in the spleens of mice treated with Ad5-OVA plus 5-FU over mice treated with Ad5-OVA alone (Fig. 2*A*). Whilst in the lymph node (Fig. 2*B*) a similar trend was observed, albeit not statistically significant. The increase observed in the lymph node for the combinatorial treatment was highly significant when compared to naïve mice, whilst either treatment alone was not. To determine if the observed increases in OVA-specific CD8^+^ T cells were dependent on the presence of the E.G7 tumor, groups of mice, not challenged with E.G7 cells, were vaccinated with Ad5-OVA (s.c.) +/−5-FU (i.p.). Subsequently, on days 7, 15 and 21, these mice were bled and their levels of OVA-specific CD8^+^ T cells in the peripheral blood were determined. Results revealed there to be no significant differences between mice vaccinated with Ad5-OVA and mice vaccinated with Ad5-OVA plus 5-FU (Fig. 2*C*).

### The Effect of 5-FU on Tregs in the Spleen and Draining Lymph Node

**Figure 3:**
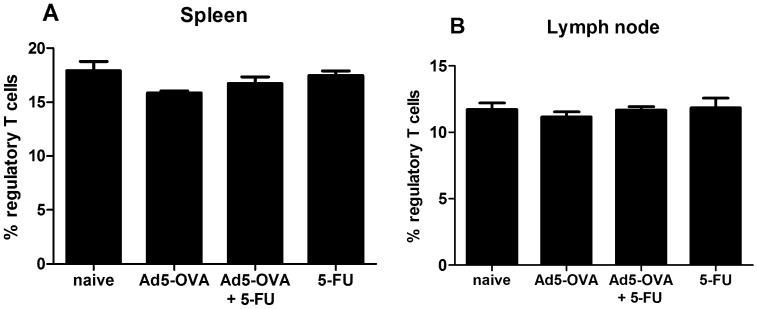
Treg levels in spleen and draining lymph node. C57BL/6 mice were challenged with E.G7 and then, 7 days post tumor challenge, were treated as indicated. On day 5 post treatment the levels of Tregs (Foxp3^+^), as a percentage of total CD4^+^CD3^+^ cells, was determined using direct immunofluorescence (see materials and methods for further details) on the samples from the spleen (*A*) and the lymph node *(B*). Results displayed are from one representative experiment (n = 4 mice per group). These results were reproducible. Using an ANOVA one way analysis of variance (with Tukey post-test) no statistically significant differences were observed in independent experiments nor after pooling of the data. Error bars represent standard error of the mean.

Tumor-challenged mice were treated as described above and on day 5 post treatment, mice from each treatment group were sacrificed and the cells from the spleen and draining lymph node were stained, using direct immunofluorescence, for the presence of Tregs as defined by their co-expression of CD3, CD4 and Foxp3. Fig. 3 shows data from one representative experiment. In the spleen the proportion of Tregs decreased marginally, but not significantly, for all treatment groups when compared to the naïve group (Fig. 3*A*). In the draining lymph there were barely any differences between groups in the proportion of CD4^+^ lymphocytes that were Tregs (Fig. 3*B*).

### The Effect of 5-FU on MDSC Levels in the Spleen and Draining Lymph Node

**Figure 4:**
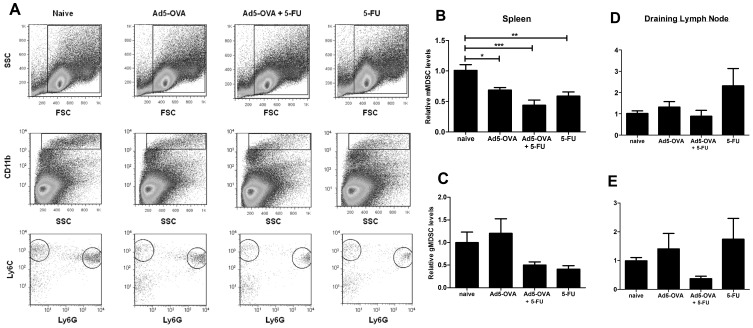
Levels of gMDSCs and mMDSCs in the spleen and draining lymph node. C57BL/6 mice were challenged with E.G7 cells and then, 7 days post tumor challenge, were treated as indicated and then 5 days later, spleen and draining lymph nodes were harvested and stained using direct immunofluorescence for detection mMDSCs and gMDSCs and then analyzed using flow cytometry (see materials and methods for further details). *A*. Example of FloJo-generated dot-plots showing how mMDSCs and gMDSCs were delineated in samples obtained from the spleen. Total splenocytes were gated using a forward scatter (FSC) versus side scatter (SSC) dot-plot (*top row*). These cells were further gated to select for CD11b^+hi^ cells using a SSC versus CD11b dot-plot (*middle row*). Then these CD11b+hi cells were further defined using a Ly6G versus Ly6C dot-plot (*bottom row*). mMDSCs were defined as CD11b^+^Ly6G^−^Ly6C^+hi^ (*circled (left side)*) and gMDSCs were defined as CD11b^+^Ly6G^+^Ly6C^+low^ (*circled (right side)*). A similar method of analysis was used for cells obtained from the draining lymph node. *B–E*. On day 5 post treatment, the levels of mMDSCs (*B* and *D*) and gMDSCs (*C* and *E*), as a percentage of total cells, were determined, in both the spleen (*B* and *C*) and the draining lymph node (*D* and *E*). Mean percentage values (and range) for the naïve groups prior to normalization were as follows: mMDSCs in the lymph node = 0.24% (range: 0.02–0.84%); mMDSCs in the spleen = 0.26% (range: 0.02–0.63%); gMDSCs in the lymph node = 0.03% (range: 0.01–0.08%); gMDSCs in the spleen = 1.35% (0.87–4.1%). Results displayed are derived from pooled data from four separate experiments, where the number of mice/treatment group in each experiment was n = 1, n = 2, n = 2, n = 4 and therefore a total of n = 9 mice from each pooled treatment group were analyzed. Statistical significance was determined using an ANOVA one way analysis of variance (with Tukey post-test) (*P<0.05, **P<0.01, ***P<0.001)). Error bars represent standard error of the mean.

Tumor-challenged mice receiving various treatments, described above, were sacrificed on day 5 post-treatment. Cells from the spleen and the draining lymph node were stained, using direct immunofluorescence, for the presence of MDSCs as defined by their expression of CD11b, Ly6C and Ly6G. MDSCs were defined as granulocytic (gMDSCs) if they were CD11b^+^Ly6C^+low^Ly6G^+^, and monocytic (mMDSCs) if they were CD11b^+^Ly6C^+hi^Ly6G^−^. Fig. 4*A* illustrates how mMDSCs and gMDSCs were delineated in samples obtained from the spleen (a similar procedure was used for the draining lymph node). Fig. 4*B–D* shows data from four pooled independent experiments. In the spleen, treatment with Ad5-OVA plus 5-FU or 5-FU alone resulted in substantial reductions (approximately 50%) of mMDSCs (Fig. 4*B*) and gMDSCs (Fig. 4*C*) compared to untreated mice, albeit not statistically significant for gMDSCs when applying an ANOVA one way analysis of variance. In the draining lymph node, none of the treatment groups displayed substantial decreases in mMDSCs (Fig. 4*D*). However, gMDSC levels in the lymph node (Fig. 4*E*) were substantially, but not significantly, decreased in mice treated with Ad5-OVA plus 5-FU compared to the naïve group. Thus the combination of Ad5-OVA plus 5-FU appeared to consistently reduce gMDSC levels in both the spleen and draining lymph nodes, and although not statistically significant, the magnitude of these reductions was, on average, quite high (≥50%). Inexplicably, levels of both mMDSCs and gMDSCs varied dramatically in the lymph nodes of mice treated with 5-FU only. We did not look for MDSC levels within the tumors themselves because the time for maximal depletion of MDSCs (day 5 post-treatment [23]) coincided with a period of substantial tumor regression resulting in very little or no tumor mass to harvest. A similar explanation also applies for why we did not assess intratumoral levels of Tregs and OVA-specific T cells (above).

**Figure 5:**
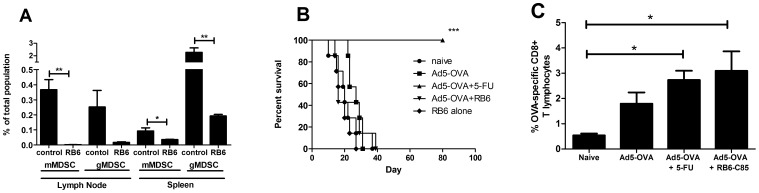
Effect of depleting MDSCs from variously vaccinated tumor-bearing mice on survival and tumor-specific T lymphocyte responses. *A*. Tumor-bearing mice were treated with multiple i.p. doses of RB6-8C5 (as described in materials and methods section) and draining lymph node and spleen cells were isolated after 6 days of depletion (day 12 post-tumor challenge) and stained for the presence of gMDSCs (CD11b^+^Ly6C^+low^Ly6G^+^) and mMDSCs (CD11b^+^Ly6C^+hi^Ly6G^−^). Data is presented as a percentage of total lymph node or spleen populations. Student T-test was used to determine statistical significance. *B.* Survival analysis: C57BL/6 mice were challenged with E.G7 and then, 7 days post tumor challenge, were treated as indicated. Survival curve represents pooled data from 2 independent experiments where a total of n = 8 mice/treatment group were used. Statistical analysis (Log-rank (Mantel-Cox) test) of survival data revealed that only the Ad5-OVA plus 5-FU treatment to be significantly different from all other treatments (*** P<0.001). *C.* Two weeks post-treatment the levels of OVA-specific CD8^+^ T cells, as a percentage of total CD8^+^CD3^+^ cells, was determined using a fluorescently-tagged OVA tetramer (see materials and methods for further details), in PBLs. Results displayed represent pooled data from 2 independent experiments and were performed in conjunction with the survival studies (see above (*B*.)). Statistical significance was determined using an ANOVA one way analysis of variance (with Tukey post-test) (*P<0.05). All error bars represent standard error of the mean.

Since the presence of 5-FU resulted in dramatically increased survival and cure rates of mice treated with Ad5-OVA we chose to investigate if it is primarily the capacity of 5-FU to deplete MDSC levels that is responsible for increased survival. In order to explore this we depleted mice of MDSCs using a Ly6G/Ly6C-specific monoclonal antibody (RB6-8C5) in a study where tumor-challenged mice were treated with Ad5-OVA +/− RB6-8C5. We found that RB6-8C5 was capable of depleting mMDSCs and gMDSCs in both spleens and lymph nodes of tumor-challenged mice (Fig. 5*A*). A survival plot (Fig. 5*B*) compares the survival of tumor-challenged mice treated with Ad5-OVA plus 5-FU versus treatment with Ad5-OVA plus RB6-8C5 and clearly shows that RB6-8C5 cannot substitute for 5-FU in terms of increasing survival rates. The reported capacity of RB6-8C5 to deplete sub-populations of dendritic cells and CD8^+^ T cells could potentially impact negatively on any therapeutic benefit that RB6-8C5-mediated MDSC depletion may confer and therefore one should be cautious in making exclusive interpretive conclusions [32], [33]. In order to at least partially alleviate concerns that RB6-8C5 treatment may have negatively impacted on the anti-OVA immune response generated by Ad5-OVA we assayed peripheral blood lymphocytes for levels of OVA-specific CD8^+^ T cells in the various treatment groups. Figure 5*C* shows results from two pooled experiments where mice treated with Ad5-OVA plus 5-FU or Ad5-OVA plus RB6-8C5 had significantly higher levels of OVA-specific T lymphocytes than the naïve mice.

## Discussion

The studies described here show that combining a single low-dose chemotherapeutic agent, 5-FU, with a therapeutic adenoviral vaccination (Ad5-OVA) results in a dramatically enhanced cancer cure rate (95%) over mice treated with either Ad5-OVA alone (7%) or 5-FU alone (30%). These results by themselves are exciting in that they record therapeutic synergy, in terms of survival, between an adenoviral cancer vaccine and low-dose chemotherapy. There have been few preclinical reports that we are aware of that have studied the therapeutic effect of combining viral cancer vaccines (encoding a TAA) and low-dose chemotherapy. Sierro et al used a recombinant lentiviral vector expressing a melanoma-specific antigen, trp-2, in combination with low-dose cyclophosphamide, which was found to have modest therapeutic properties but nevertheless exhibited synergy [25]. Ko et al showed that the combination of an adenovirus encoding human (xenogeneic) Her-2/neu, a low-dose chemotherapeutic gemcitabine (also known to deplenish MDSCs) and the Treg suppressive antibody, anti-GITR, was capable of causing tumor regressions in a murine colon cancer model where the cancer cell line used was transfected with syngeneic Her-2/neu [34].

In our studies, analysis of OVA-specific CD8^+^ T cell levels in the spleen and draining lymph node revealed increased levels (approx. ≥2-fold) in mice treated with Ad5-OVA plus 5-FU versus mice vaccinated with Ad5-OVA alone, whilst mice treated with 5-FU alone had no detectable increase over levels seen in naïve mice (Fig. 2*A and B*). This trend was also observed in the peripheral blood (Fig. 5*C*) of tumor-challenged mice. Of particular interest was the finding that no such increase occurred when mice were similarly vaccinated in the absence of tumor-challenge (see Fig. 2*C*). That the increase in OVA-specific CD8^+^ T cells is tumor-dependent suggests the possibility that 5-FU, aside from its ability to effectively reduce tumor bulk, may be contributing to the therapeutic benefit by enhancing the Ad5-OVA induced anti-tumor immune response. There are a number of not necessarily mutually exclusive ways that 5-FU may be achieving this. The first possibility is that 5-FU, through the induction of tumor cell death, generates a massive tumor antigen load for local dendritic cells to engulf and present to T lymphocytes in the draining lymph node. This combined with the potent capacity of Ad5 to stimulate dendritic cells could be a primary, or contributory, factor explaining the increase in OVA-specific CD8^+^ T cells and the resulting benefits to survival [9]. A second possibility is that 5-FU may be promoting a more potent effector response through the diminution of MDSC populations. It has recently been reported that low concentrations of 5-FU were capable of causing immune-dependent regression of EL4 tumor cells and that this effect was suggested to be at least partially due to the capacity of 5-FU to deplete tumors and spleens of MDSC populations [23]. MDSCs are a heterogeneous population of myeloid cells that accumulate in tumors and secondary lymphoid organs of tumor-bearing individuals and suppress T lymphocyte responses [24], [35]. MDSCs have been recently subcategorized into two major groups, granulocytic MDSCs (gMDSCs) which are CD11b^+^Ly6G^+^Ly6C^low^, and monocytic MDSCs (mMDSCs) which are CD11b^+^Ly6G^−^Ly6C^high^ [36]. We observed that both of these populations decreased in the spleens of mice treated with Ad5-OVA plus 5-FU, or 5-FU alone (Fig. 4*B and 4C*). Although it should be noted that it was only the reduction of mMDSC levels in the spleen that proved to be statistically significant. To investigate the degree to which MDSC depletion may contribute to enhancing OVA-specific CD8^+^ T cell levels and increasing survival of Ad5-OVA-treated mice we combined Ad5-OVA therapy with antibody (RB6-8C5)-mediated MDSC depletion. RB6-8C5 is a monoclonal antibody with high affinity for Ly6G, and to a lesser extent Ly6C, which has been used to deplete mice of MDSCs, although it should also be noted that neutrophils are also substantially depleted using this antibody [37]. We found that gMDSC and mMDSC depletion was effectively achieved, using RB6-8C5, in both the spleens and lymph nodes of tumor-challenged mice (Fig. 5*A*). When Ad5-OVA was combined with RB6-8C5 the increase in OVA-specific CD8^+^ T cells, relative to Ad5-OVA alone, mirrored that seen for Ad5-OVA plus 5-FU (Fig. 5*C*). This finding indirectly suggests that 5-FU may be contributing to higher numbers of OVA-specific CD8^+^ T cells through the reduction of MDSCs. Alternatively, this finding may be purely circumstantial and 5-FU may be enhancing OVA-specific CD8^+^ T cell responses irrespective of its capacity to reduce MDSC levels. No increase in survival time was noted for mice treated with Ad5-OVA plus RB6-8C5 compared to mice treated with Ad5-OVA alone (Fig. 5*B*). Thus, whilst the reduction of MDSCs may potentially explain the increased OVA-specific response observed when Ad5-OVA is combined with 5-FU, it does not explain the dramatic increase in survival times that this combination affords. It would be therefore tempting to conclude that MDSC depletion does not contribute to increased survival, however, care must be taken in making this interpretation given that RB6-865 has been shown capable of reducing subpopulations of dendritic cells [33] and CD8^+^ T cells [32] in addition to its desired capacity to deplete MDSCs. However, it has recently been shown that RB6-8C5 could deplete MDSCs in a murine lung cancer model and result in enhanced CTL and antigen presenting cell activity as well as enhanced vaccine-mediated tumor regression [38]. A third potential way that 5-FU may be enhancing immune-mediated survival derives from findings that 5-FU can increase Fas on tumor cells making them more susceptible to CTL-mediated apoptosis [39]. Thus it is possible that 5-FU can prime tumor cells for immune attack by altering expression of key surface proteins, such as Fas and possibly others (e.g. Trail and MHC class I), and in this way contribute to enhancing Ad5-OVA anti-tumor immunity. The possibility that 5-FU is inducing an immunogenic tumor cell death/apoptosis is not likely given that it has been previously shown that treatment of EL4 cells (a non-OVA expressing predecessor to E.G7) with 5-FU did not increase surface expression of calreticulin, nor were the anti-tumor effects of 5-FU found to be TLR4-dependent [23].

We found that 5-FU alone caused tumor regression in all mice and that 30% of these mice remained tumor-free through the course of the experiments (>80 days). Based on findings by others that EL4 tumor regression induced by 5-FU was only observed in immunocompetent and not in nude mice, it is possible that 5-FU alone may mediate its curative effect, observed in our studies, at least partially through adaptive immunity [23]. However, this appears unlikely to be the situation in the experiments performed here for two main reasons. Firstly, 5-FU alone was incapable of inducing detectable levels of OVA-specific CD8^+^ T cells in tumor-bearing mice. Secondly, mice that were treated with 5-FU alone and became tumor-free for greater than 100 days, which we defined as “cured”, were not resistant to a subsequent rechallenge with E.G7 (data not shown). This was in contrast to those mice that were cured through treatment with Ad5-OVA alone or Ad5-OVA plus 5-FU, which were resistant to subsequent E.G7 rechallenge. Therefore, if mice treated with 5-FU alone mediate tumor rejection through immune mechanisms, it is more likely to be through NK cells than an adaptive T lymphocyte response.

In terms of translation into the clinic, a limitation of the tumor model used here is that it involves a xenogeneic model tumor antigen and ideally a tolerogenic model would ultimately be preferable. It is important to note that supplementary treatments may also be required for cancer patients, such as the further dampening of the suppressive arms of the host’s immune system, since breaking tolerance to TAAs will prove more difficult than in our model system. This is highlighted by the findings of Ko et al, mentioned above. Further studies in mouse cancer models where the mice are tolerized to the TAA are a high priority for establishing further the potential clinical relevance of the treatment regime described here.

There is a growing awareness among clinical oncologists and tumor immunologists for the need to exploit the potential therapeutic synergy of combining cancer vaccines with other more conventional forms of therapy such as chemotherapy [14]. The motivation for exploring such combinations is the potential for increased survival rates and decreased side effects. Low-dose chemotherapy in combination with active cancer vaccines has previously shown promise in preclinical therapeutic settings [25], [40]. Although there have been clinical trials combining cancer vaccines with chemotherapy, these have, with rare exceptions, usually not been strategically designed to favor immune therapy since they have involved high-dose chemotherapy and often involved chemotherapeutic drugs that have no established capacity at diminishing the suppressive arm of the immune response [41], [42]. Here we show convincing evidence of the therapeutic benefit of combining a low-dose chemotherapeutic drug, capable of diminishing MDSC levels, with an adenoviral vaccine encoding a model TAA. These findings illustrate the potential promise for such a treatment strategy in a clinical setting.
